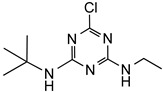# Characterization of MS/MS Product Ions for the Differentiation of Structurally Isomeric Pesticides by High-Resolution Mass Spectrometry

**DOI:** 10.3390/toxics6040059

**Published:** 2018-10-02

**Authors:** Alberto Nuñez, Yelena Sapozhnikova, Steven J. Lehotay

**Affiliations:** US Department of Agriculture, Agricultural Research Service, Eastern Regional Research Center, 600 East Mermaid Lane, Wyndmoor, PA 19038, USA; Yelena.Sapozhnikova@ars.usda.gov (Y.S.); steven.lehotay@ars.usda.gov (S.J.L.)

**Keywords:** high-resolution mass spectrometry, Orbitrap, structure elucidation, pesticide residue analysis

## Abstract

Structural isomeric pesticides are used in agriculture and may be challenging to differentiate for accurate identification in pesticide monitoring programs. Due to structural similarity, isomeric pesticides are difficult to separate chromatographically, and thus, their accurate identification may rely solely on mass spectrometric analysis (MS). In this study, we challenged the ability of high-resolution quadrupole-orbitrap (Q-Orbitrap) mass spectrometry to produce and evaluate the tandem mass spectrometry (MS/MS) product ions for the selected five pairs of isomeric pesticides from different classes: Pebulate and vernolate, methiocarb and ethiofencarb, uniconazole and cyproconazole, sebuthylazine and terbuthylazine, and orbencarb and thiobencarb. The use of Q-Orbitrap instrument with a mass error <3 ppm allowed proposed elucidation of the product ion structures with consideration of the ion formulae, data interpretation, and literature searches. Product ions unique to pebulate, vernolate, methiocarb, ethiofencarb, and uniconazole were observed. Elucidation of the observed MS/MS product ion structures was conducted, and the fragmentation pathways were proposed. This information is valuable to increase selectivity in MS/MS analysis and differentiate isomeric pesticides, and thereby reduce the rates of false positives in pesticide monitoring programs.

## 1. Introduction

Numerous pesticides are used in agriculture worldwide to protect crops and increase harvest yields, with over 1.1 billion pounds used annually in the USA, and nearly six billion pounds (three million tons) usage worldwide [1]. Use of specific pesticides varies by different countries, and certain pesticides may be approved for applications in some countries but banned or no longer used in others. To protect consumer health, maximum residue limits (MRLs), or tolerances in the USA, are established and enforced by regulatory agencies around the world. MRLs depend on pesticide toxicity, physico-chemical properties, and application rates, which can range significantly for the same pesticide in different commodities [2]. Additionally, different countries may have different MRLs for the same pesticide in the same commodity. Hence, reliable identification and accurate measurement of pesticide residues in foods is important in global food trade because false positives and other forms of incorrect results can have detrimental economic and health consequences. 

Common approaches for analysis of pesticide residues include liquid chromatography (LC) or gas chromatography (GC) with single or triple quadrupole for tandem mass spectrometry (MS) for separation and identification of targeted contaminants. In recent years, however, high-resolution accurate mass (HRAM) analyzers are becoming more common in routine analysis of pesticides and other contaminants [3,4]. During the MS/MS method development phase, precursor, and fragment/product ions are selected for analyte identification, and often, the most intense ions are selected. Many pesticides belonging to the same chemical classes (triazines, carbamates, *etc.*) produce the same product ions, and analyte/analyte interferences commonly complicate their accurate quantification. To achieve better selectivity and increase confidence in identification, specific fragments should be selected and studied when possible.

The use of HRAM analyzers in comparison with low resolution quadrupole analyzer instruments facilitates ion mass determination with error <5 ppm required for ion formula determination [5]. Combining this information with literature searches, databases, and data interpretation helps with the assignment of product ions structures. Currently, few published manuscripts on the analysis of contaminants report fragment/product ions with elucidation of the corresponding structures [6,7,8,9,10]. Yet, elucidation of ion structures is important to provide further support for elimination of false positive findings. This becomes even more important when dealing with identification of structurally isomeric pesticides, i.e., pesticides with the same molecular formula and weight, but different atom arrangement. 

One example of isomeric pesticides are the carbamate herbicides, orbencarb and thiobencarb. Orbencarb (*S*-(2-chlorobenzyl) *N,N*-diethylthiocarbamate) was previously used on cereals, including barley, wheat, rye, maize, soybean, *etc.*, but not currently authorized for use in the European Union (EU) and not registered for use in the USA. Thiobencarb (*S*-(4-chlorobenzyl)-*N,N*-diethylthiocarbamate) differs from orbencarb only by Cl positioning on benzene ring (Table 1). It is used for weed control in paddy fields, mostly on rice, and according to Pesticide Action Network of North America, has an active status in the US products [11], but it is not approved for use in the EU. The MRL for thiobencarb is 10–200 ng/g depending on the country and commodity. As isomeric pesticides possess very similar properties, they are usually difficult or impossible to separate chromatographically. For example, both orbencarb and thiobencarb had the same retention time of 19.38 min and the same ions at *m/z* 258, 125, and 100 when analyzed by LC-MS/MS [12]; thus they were not differentiated by either LC separation or MS identification. MS/MS spectra of isomeric compounds are usually very similar and may have few distinct ions to tell them apart. In order to support these differentiations, it is important to show structures for those specific ions in support of the method of analysis. The use of HRAM is essential for the determination of ion formulas to be able to determine structures and fragmentation pathways [13,14]. 

In this study, we selected five pairs of isomeric pesticides (see Table 1): Pebulate and vernolate, methiocarb and ethiofencarb, uniconazole and cyproconazole, sebuthylazine and terbuthylazine, and orbencarb and thiobencarb, to test the hypothesis of applying HRAM for finding unique product ions to distinguish between the isomers. A Q-Orbitrap HRAM MS instrument with an error <3 ppm was utilized for identification of products ion formulae and the proposal of structures and pathways of fragmentation to differentiate isomeric pesticides. When necessary, the fragmentation patterns were based on product ions obtained from the MS^2^ and their selected ions spectra (MS^3^) obtained with a triple quadrupole instrument with a linear trap (Q-Trap) mass spectrometer.

## 2. Materials and Methods

### 2.1. Reagents

Positive ion calibration solution for the Q-Orbitrap instrument was from Thermo Scientific (Rockford, IL, USA). Pesticide standards (purity > 95–99%) and formic acid (purity 98%) were obtained from Sigma Aldrich (St. Louis, MO, USA). Acetonitrile (MeCN), methanol (MeOH) and water were Optima LC-MS grade obtained from Fisher Scientific (Phillipsburg, NJ, USA), and deionized water was obtained from a Millipore (Bedford, MA, USA) Milli-Q system. Stock standard solutions were prepared at 2 mg/mL in MeOH, and working solutions at 0.5 μg/mL were prepared from stock solutions in 1:1 MeCN:H_2_O (*v*:*v*) containing 0.1% formic acid.

### 2.2. Instrumentation and Conditions

Pesticide standard solutions (0.5 μg/mL) were infused at 10 µL/min using a Chemyx syringe pump model Fusion 101 into a Q-Exactive Plus (Thermo Scientific, Madison, WI, USA) mass spectrometer equipped with the electrospray ionization (ESI) probe (HESI-II) in positive mode to obtain MS/MS spectra. Collision energies were optimized and precursor ions were obtained with the mass error of <3 ppm. Capillary temperature was 300 °C with a spray voltage of 3.5 kV and nitrogen as sheath gas set to 60 arbitrary units of the HESI probe. Resolution was set to 140,000 (FWHM) for MS and MS/MS using normalized collision energy (NCE) adjusted to produce the ions of interest. The collision induced dissociation (CID) at the ion source was set at 5 V, except when a specific fragment ion was produced for subsequent MS/MS, in which case the CID was set to 30 V. The instrument was calibrated in the positive mode with a standard solution (Thermo Scientific) and the polysiloxanes *m/z* of 371.10124 and 445.12003 were used as lock masses in the MS mode.

Ion formation pathways were also investigated by infusion the selected pesticide standard solutions using a Sciex 6500 Q-Trap mass spectrometer (Sciex, Framingham, MA, USA) to obtain MS^2^ and MS^3^ spectrum of specific ions of interest. Ion source parameters were: Curtain gas at 10 psi, the ion spray potential was 5,000 V, the source temperature was 350 °C, entrance potential 10V, collision gas (CAD) was set at medium, and ion source gases were 12 psi (GS1) and 10 psi (GS2).

## 3. Results and Discussion

Extensive compiled literature on mechanisms of fragmentation is available for electron ionization, also referred as odd electron ionization, but less is found regarding more generalized soft ionization, or even electron ionization, producing protonation, deprotonation, or alkyl adduct formation of the analytes. Recently, a very comprehensive compilation of drugs and pesticides MS/MS data interpretations has been published by Niessen and Correa [10], providing an excellent source for structural identification of MS/MS ions resulting from soft ionization mass spectrometry. In addition, availability of commercial software for spectra interpretation and public compound databases can help in elucidation of structures of product ions, but this approach should be considered with caution and some of these limitations have been described by Wright et al. [15]. Previously, we conducted structural characterization of product ions for 120 veterinary drugs with ESI quadrupole time-of-flight (Q-TOF) MS [7,8,9]. In the present study, a Q-Orbitrap instrument was used for characterization of product ions to differentiate isomeric pesticides. Occasionally, to better understand fragmentation pathway, product ions were selected for additional fragmentation. This was achieved by using a Q-Trap to obtain the MS^3^ spectrum or, alternatively, by using adequate collision dissociation (CID) energy at the ion source of Q-Orbitrap to generate fragmentation for selection and subsequent MS/MS fragmentation. During the discussion, and with the purpose of simplification, the ion masses are used without considering the fractional masses that are reported in figures and schemes.

### 3.1. Pebulate and Vernolate

**Figure 1:**
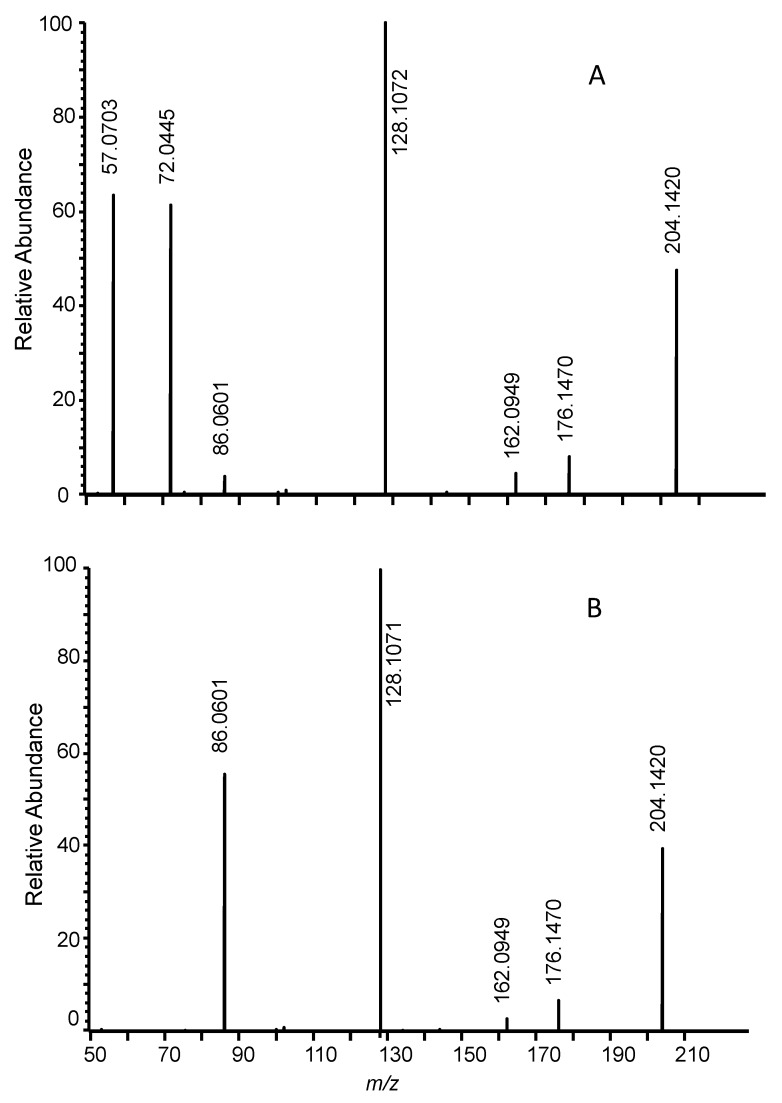
Positive ESI spectra obtained with an NCE 20 eV for: (**A**) pebulate and (**B**) vernolate.

Vernolate (*S*-propyl-*N,N*-dipropylcarbamothioate) and pebulate (*S*-propyl-*N*-butyl-*N*-ethylcarbamothioate) are thiocarbamate herbicides. Both pesticides’ applications ceased in the USA since 2001 and 2009, respectively [16], and in the EU in 2009 [17]. These pesticides are *S*-propyl thiocarbamates bearing a *N*-butyl-*N*-ethyl group (pebulate) and a *N,N*-dipropyl group (vernolate) substituent at the nitrogen. MS/MS spectra for these pesticides are presented in Figure 1, showing a very similar profile. The structure of some of these ions have been previously reported [18] and more recently described by Neissen and Castro [10]. The spectrum in Figure 1A for pebulate has two product ions at *m/z* 57 and 72 that are not observed for vernolate (Figure 1B). Additionally, vernolate has a significant ion at *m/z* 86 that is small for pebulate.

**Scheme 1:**
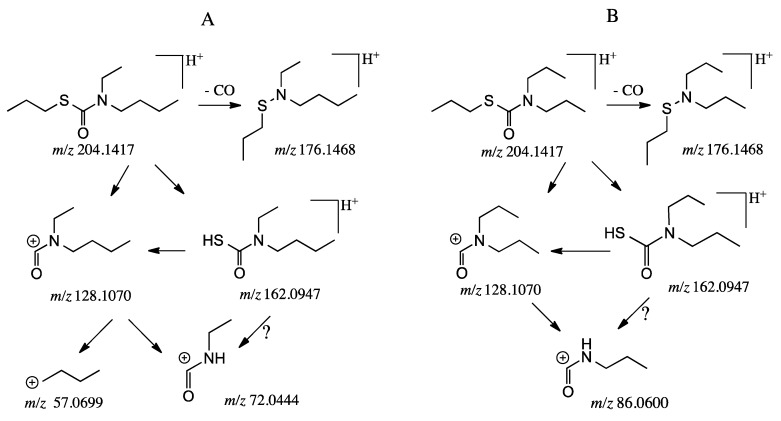
Proposed fragmentation pathway for (**A**) pebulate and (**B**) vernolate.

Scheme 1 is the proposed fragmentation pathway for these pesticides indicating formation of two ions at *m/z* 128 and 162 that lead to product ions at *m/z* 57 and 72 for pebulate, and product ion at *m/z* 86 for vernolate. This ion resulted from the loss of butyl group but the equivalent ion resulting from the loss of ethyl group was not observed in the spectra. It is not clear if ion at *m/z* 162 can lead to those product ions, and is thus shown with a question mark in Scheme 1.

**Figure 2:**
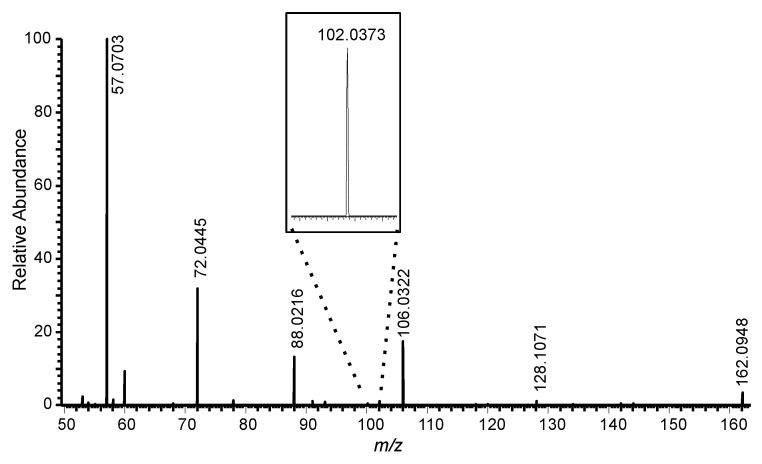
CID/HCD MS/MS spectrum of the selected ion at *m/z* 162 product ion of pebulate. The insert is the zoom region showing ion at *m/z* 102.

**Scheme 2:**
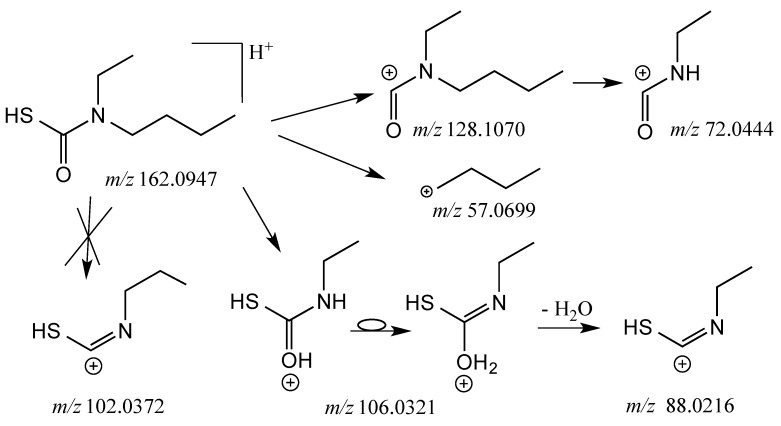
Proposed fragmentation pathway for the CID generated product ion at *m/z* 162 of pebulate.

The small ion at *m/z* 86 in the spectrum of pebulate is difficult to explain based on the fragmentation pattern observed. To further verify its origin, the *m/z* 162 ion was generated by adjusting CID energy at the ion source of Q-Orbitrap, and then selected for MS/MS. The resulting spectrum is shown in Figure 2. This approach generates a set of product ions that are presented in the pathway in Scheme 2. A careful inspection of the spectrum indicated the presence of a small ion at *m/z* 102 (Figure 2 insert). This ion is not expected for pebulate, nor is *m/z* 86 product ion, and consequently, this suggests that pebulate was contaminated with vernolate. This approach is not a true MS^3^ because other isobaric ions could be present in the ion source, thus has to be used with caution, especially when low mass ions are selected. Additionally, the inclusion of solvent clusters in MS/MS high-energy collision cells can produce misleading product ions. However, exceptional mass accuracy and resolution of Q-Orbitrap are highly beneficial for assignments of ion formulae to better interpret fragments observed in this manner.

### 3.2. Methiocarb and Ethiofencarb

Methiocarb (3,5-dimethyl-4-methylthiophenyl-*N*-methylcarbamate) is a carbamate pesticide, currently approved for use in the EU. It is used for seed treatment, and applied to various agricultural crops, such as maize, lettuce, and fruits. It has an EU MRL of 50 ng/g in food of animal origin, 100 ng/g in grains, and 100–1000 ng/g in fruits and vegetables [17]. Methiocarb does not appear to be used in the USA, and no tolerance is listed in the USA MRL global database [19]. Ethiofencarb (α-ethylthio-*o*-tolyl methylcarbamate) is not approved for use in the EU, and no longer produced or used in the USA.

**Figure 3:**
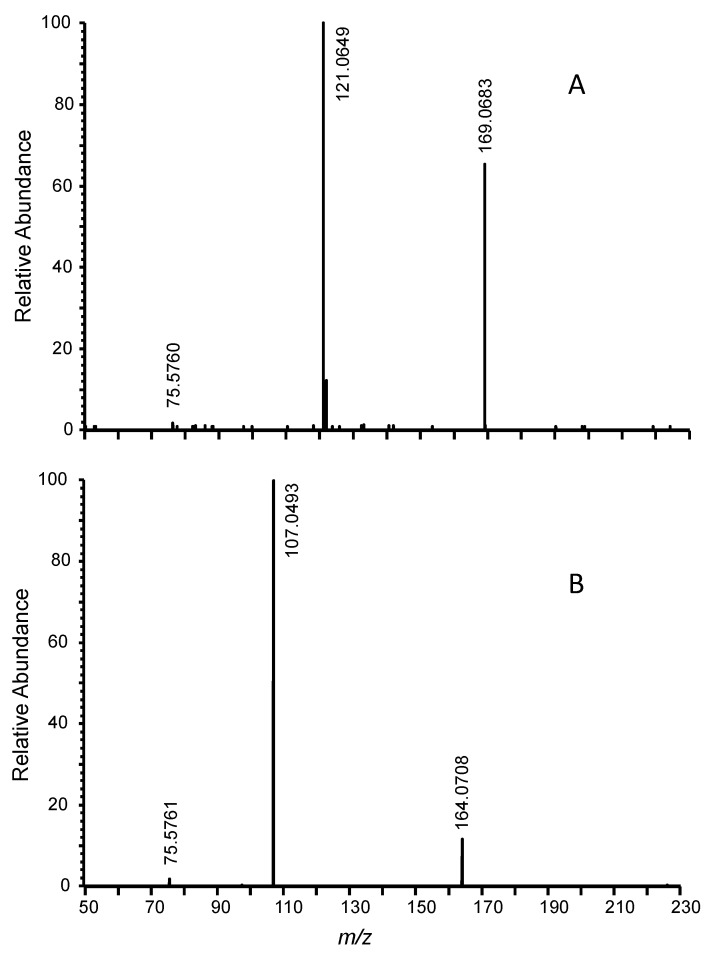
Positive ESI spectra obtained with an NCE 20 eV for (**A**) methiocarb and (**B**) ethiofencarb.

**Scheme 3:**
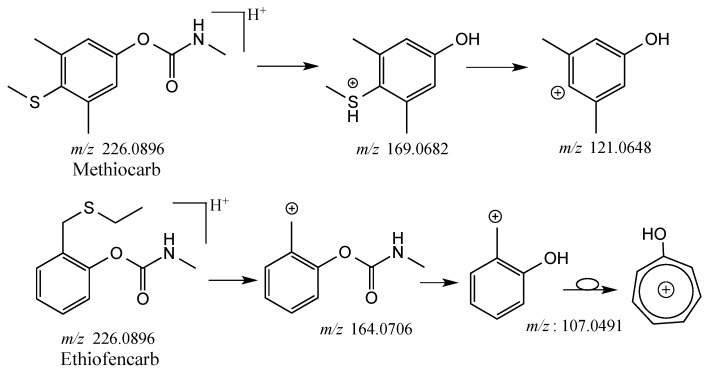
Proposed pathway for the formation of the differentiation ions for methiocarb and ethiofencarb.

These two pesticides have MS/MS spectra producing a few fragments at 40–50 eV with product ions that can clearly differentiate between methiocarb and ethiofencarb as shown in Figure 3. However, the ions at *m/z* 107 and 169 are also observed at higher collision energies and only specific ions at *m/z* 121 for methiocarb and ion at *m/z* 164 can be used for differentiation. The pathway for the formation of these product ions is shown in Scheme 3, where ethiofencarb’s loss of carbamate group leads to formation of ion at *m/z* 169 that subsequently leads to ion at *m/z* 121 after losing methanethiol [10]. Furthermore, the hydroxybenzyl cation at *m/z* 164 could rearrange to hydroxytropyllium ion [20].

### 3.3. Uniconazole and Cyproconazole

Uniconazole ((*E-*3*S*)-1-(4-chlorophenyl)-4,4-dimethyl-2-(1,2,4-triazol-1-yl)- 1-penten-3-ol) and cyproconazole (2-(4-chlorophenyl)-3-cyclopropyl-1-(1H-1,2,4-triazol-1-yl)-2-butanol) are triazole fungicides. Uniconazole is not approved for use in the EU, but is used in the USA as plant growth regulator for flowering plants and vegetables (such as tomatoes, peppers, cucumber, and avocado) with the US tolerance of 10 ng/g. Cyproconazole, on the other hand, is approved for use in the USA and EU, and is widely used as a fungicide for cereals, vegetables and fruits, and nuts. Its MRL ranges from 10–1000 ng/g depending on the country and commodity.

**Figure 4:**
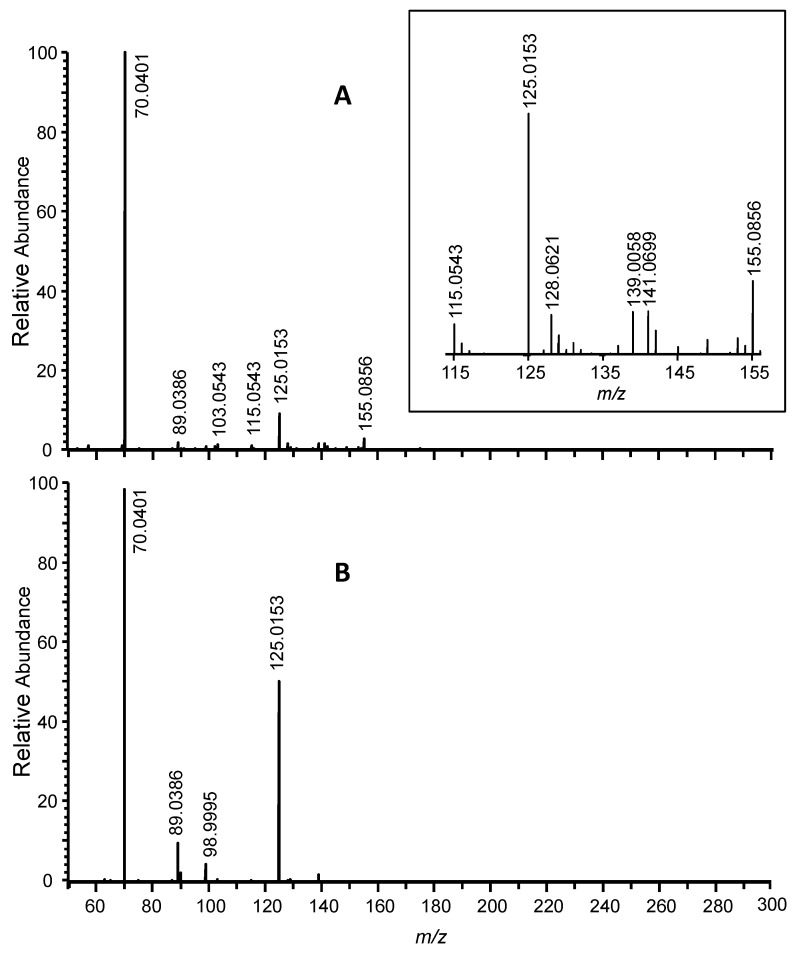
Positive ESI spectra obtained with an NCE 90 eV for: (**A**) uniconazole, with insert expanding the mass range between 115 and 155, and (**B**) cyproconazole.

In terms of chemical structure, cyproconazole and uniconazole are triazolyl derivatives with a monochlorobenzyl group as shown in Table 1. The most significant fragments for these compounds have been described by Niessen and Correa [10] and fragments include the loss of the triazolyl and the product ion corresponding to its protonated form ([C_2_H_4_N_3_]^+^). Another significant fragment is the chlorobenzyl ion. Ions like these have been reported as the tropylium cation, but rather high energy is needed to rearrange to this configuration and it formation is questioned [21,22]. Figure 4 shows the spectra for uniconazole and cyproconazole with product ions at *m/z* 70 for the protonated triazolyl cation and *m/z* 125 for the monochlorobenzyl cation. The spectra require 90 eV to produce ions that can be used to differentiate both monochlorobenzyl derivatives. Under this condition, loss of water is not observed, but this loss occurs at lower energy, most likely because the preferred site of protonation is at the triazolyl ring, leading to the elimination of this group (*m/z* 70).

**Scheme 4:**
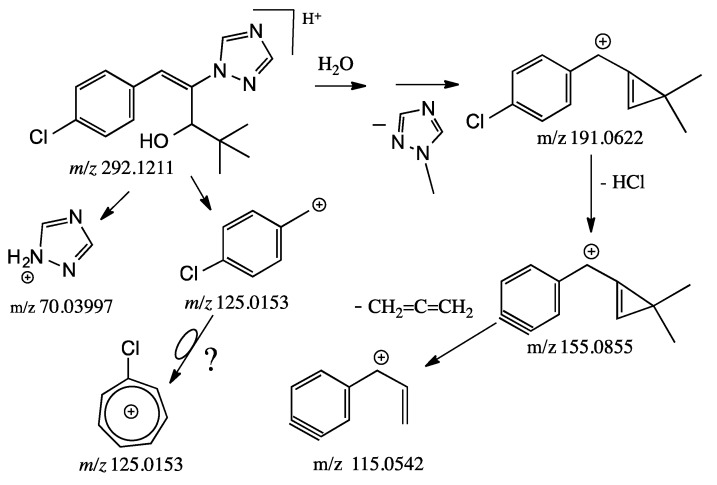
Proposed pathway for the formation of the differentiation ions for uniconazole.

Both spectra in Figure 4 have the same fragment peaks at different intensities, but uniconazole presented two distinctive product ions at *m/z* 155 and 115 at 70–90 eV that were not found for cyproconazole (Figure 4A insert). On the other hand, cyproconazole did not show any characteristic fragments. Q-Trap isolation and fragmentation of product ion at *m/z* 155 confirmed the subsequent ion at *m/z* 115. The fragmentation pathway required to explain these ions is complex and requires elimination of water, methyl, and triazole groups. The proximity of triazole to the methyl moiety in the terbuthyl group in a 3D model suggests that the methyl can migrate to the triazole and be eliminated as methyl triazole, leading to the ion at *m/z* 191, as shown in Scheme 4. The latter ion is very small and requires less energy to be observed, but seems to lead to the formation of product ion at *m/z* 155 after the loss of HCl. Positive charge in the structure will provide resonance stability to the ion. Loss of 1,2-propadiene from ion at *m/z* 155 produced a product ion at *m/z* 115. These two ions appear to be specific for identification of uniconazole, however, no specific ions were found for cyproconazole to differentiate it from uniconazole.

### 3.4. Orbencarb and Thiobencarb

These two pesticides differ in the position of chlorine group at the benzene ring (Table 1); consequently, no specific ions were observed for their differentiation. However, they form an intense product ion at *m/z* 125 (chlorobenzyl), which after loss of HCl produces *m/z* 89 (data not shown). Due to the ortho position of Cl in orbencarb and para position in thiobencarb, the product ion *m/z* 89 has different relative ion abundance ratios for these pesticides. Additionally, a product ion at *m/z* 100 corresponding to [(CH_3_-CH_2_)_2_N-C=O]^+^ is affected by the position of Cl, thus producing different ion intensities. Under controlled conditions, fragmentation of ions at *m/z* 125 and *m/z* 100 as precursors can provide additional evidence for distinction of these pesticides. The difference in intensities depends on collision energy, but when using the Q-Trap instrument, thiobencarb showed intensities of approximately 60% and 25% for ions at *m/z* 89 at *m/z* 100, respectively. The ion at *m/z* 89 is in agreement with a chlorobenzyl ion at *m/z* 125 that did not seem to rearrange into a chlorotropyllium ion (see Scheme 4) [21] because both pesticides would have the same possibility of forming this ion at *m/z* 89.

### 3.5. Sebuthylazine and Terbuthylazine

Sebuthylazine (2-*N*-butan-2-yl-6-chloro-4-*N*-ethyl-1,3,5-triazine-2,4-diamine) and terbuthylazine (2-*N*-tert-butyl-6-chloro-4-*N*-ethyl-1,3,5-triazine-2,4-diamine) are triazine group herbicides, very similar to the well-known herbicide, atrazine. No currently registered pesticide products containing sebuthylazine were found, and it is listed as obsolete in the EU. Terbuthylazine, on the other hand, is widely used in Europe and 45 other countries to control weeds in corn, potatoes, sorghum, pea, bean, grape, fruit, citrus, etc. with MRLs of 50–100 ng/g [15]. It has become a widely used triazine herbicide in Europe since atrazine was discontinued in 2004. Conversely, terbuthylazine is not registered in the USA [16]. 

Sebuthylazine and terbuthylazine are monochlorotriazines, and other well-known herbicides from this class include atrazine, propazine, and cyanazine. Fragmentation pathway of protonated monochlorotriazines has been previously extensively investigated and described [12,23]. MS/MS ions observed for both pesticides included *m/z* 174, 104, 132, and 96, similar to the ones previously reported, but no distinct product ions for differentiation of these isomers were observed.

## 4. Conclusions

Analysis of isomeric pesticides requires the identification of product ions that are unique to each isomeric form. The use of HRAM mass spectrometry provided the molecular ion formula that led to the proposal of pathways and structure for these distinctive product ions for pebulate-vernolate, uniconazole-cyproconazole, and methiocarb-ethiofencarb. Thiobencarb and orbencarb can only be differentiated according to the relative intensities of the ions at *m/z* 89 and 100, but for sebuthylazine and terbuthylazine no differentiation could be established. The importance of identifying specific ions and determining their structures provides further support for elimination of false positives, and consequently, avoiding wrongful prosecution by providing further evidence for the analytical results in legal proceedings. 

## Figures and Tables

**Table 1:** Isomeric structures of studied pesticides.

Name	CAS Number	Formula	Exact Mass	Structure
Vernolate	1929-77-7	C_10_H_21_NOS	203.1343	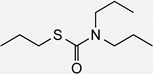
Pebulate	1114-71-2	C_10_H_21_NOS	203.1343	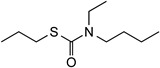
Cyproconazole	94361-06-5	C_15_H_18_ClN_3_O	291.1138	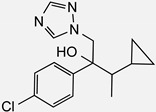
Uniconazole	83657-22-1	C_15_H_18_ClN_3_O	291.1138	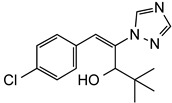
Methiocarb	2032-65-7	C_11_H_15_NO_2_S	225.0823	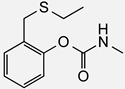
Ethiofencarb	29973-13-5	C_11_H_15_NO_2_S	225.0823	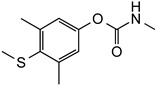
Thiobencarb	28249-77-6	C_12_H_16_ClNOS	257.0641	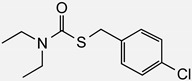
Orbencarb	34622-58-7	C_12_H_16_ClNOS	257.0641	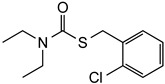
Sebuthylazine	7286-69-3	C_9_H_16_ClN_5_	229.1094	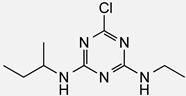
Terbuthylazine	5915-41-3	C_9_H_16_ClN_5_	229.1094